# Exploring the Effects of Geopolitical Shifts on Global Wildlife Trade

**DOI:** 10.1093/biosci/biac015

**Published:** 2022-04-06

**Authors:** Joana Ribeiro, Pedro Bingre, Diederik Strubbe, Joana Santana, César Capinha, Miguel B Araújo, Luís Reino

**Affiliations:** University of Porto, Porto, Portugal; Coimbra Polytechnic, Coimbra, Portugal; University of Ghent, Ghent, Belgium; University of Porto, Porto, Portugal; University of Lisbon, Lisbon, Portugal; University of Évora, Évora, Portugal; University of Porto, Porto, Portugal

**Keywords:** biological invasions, socioeconomic scenarios, trade barriers, law enforcement

## Abstract

International wildlife trade is a major driver of species extinction and biological invasions. Anticipating environmental risks requires inferences about trade patterns, which are shaped by geopolitics. Although the future cannot be predicted, scenarios can help deal with the uncertainty of future geopolitical dynamics. We propose a framework for generating and analyzing scenarios based on four geopolitical storylines, distinguished by combinations of international trade barrier strength and domestic law enforcement degree across countries supplying and demanding wildlife. We then use historical data on bird trade to classify countries into geopolitical profiles and confirm that trade barriers and law enforcement allow predicting bird trade patterns, supporting our scenarios’ plausibility and enabling projections for future global bird trade. Our framework can be used to examine the consequences of geopolitical changes for wildlife trade and to advise policy and legislation. Reducing demand for wildlife and ameliorating global inequality are key for curbing trade related risks.

Approximately one million species are now facing extinction because of land- and sea-use change, harvesting, invasions, pollution and climate change (WWF 2020). Internationally coordinated policies are needed for averting this global environmental crisis, but coordination is becoming challenging as the sociopolitical order dominating the world since the end of the Second World War is being challenged (O'Sullivan 2019). Free trade agreements, coupled with international capital and technological advances, led to a period of unparalleled growth in both gross domestic product and global trade from the 1950s until the early 2000s,  coinciding with the emergence of new middle classes in developing regions (Swarup 2016). However, discontent about socioeconomic outcomes of globalization has contributed to the emergence of nationalistic and populist political movements, the rise of military tensions, economic and commercial disruptions, and shifting domestic settings and international relations. All in all, these shifts are leading to a different global order (Friedman 2009, MOD 2018, O'Sullivan 2019) that often compromise pivotal environmental policies. Although the impacts of land degradation, deforestation, and climate change have recurrently been investigated together with their impacts on biodiversity (e.g., Hof et al. 2013, Symes et al. 2018), the effects of geopolitical changes are seldom examined when projecting future challenges for biodiversity conservation.

Wildlife trade for pets, luxury goods, and medicinal parts is a profitable economic activity, with billions of living organisms or derived products being traded worldwide annually (Karesh et al. 2005, Jenkins 2007, UNEP-Interpol 2016). This market is a prominent driver of vertebrate extinction (Maxwell et al. 2016) and biological invasions (Carrete and Tella 2008, Essl et al. 2015, Cardador et al. 2019), the latter often acting as a vector for new diseases (Lycett et al. 2019, Wacharapluesadee et al. 2021), changing ecosystem processes (IPBES 2019), and reducing the value of land and water for human activities (McNeely et al. 2001, Vilà et al. 2010).

Global trade, land-use change, and climate change are well-known drivers of native population declines and biological invasions. Societal aspects, such as international politics, governance, legislation, lifestyle, social norms, and technological development also have an enormous influence (e.g., Perrings et al. 2005, Lockwood et al. 2019), but their uptake into future conservation risk projections has, until recently, been low (Lenzner et al. 2019, Roura-Pascual et al. 2021). Although they are difficult to quantify, inferences about future trade patterns are required for anticipating future risks of species depletion and biological invasions. In the past, trade was constrained by geography, so it had a relatively high degree of predictability. In a globalized world, where barriers to trade are geopolitical more than geographical, inferences about trade require understanding of socioeconomic drivers and how they act in both supplying and demanding countries. Therefore, anticipating future conservation and bioinvasion risks requires careful consideration of the outcomes of alternative geopolitical scenarios.

Socioeconomic drivers and geopolitical trajectories of global wildlife trade Government policies and the rigor with which these policies are implemented strongly affect global (wildlife) trade, because trade barriers between countries and law enforcement within countries can affect the volume of international commerce. The aim and effectiveness of such policies depend on a variety of factors, including whether traded products are sold in a supply or demand dominated market situation or whether products tend to be income elastic or not (box 1; McNelly 2001). Wildlife trade is an important source of income for several cash-poor biodiversity-rich economies (Rijsoort 2000) exporting wildlife products and rising consumer income can drive increased demand for some products, as is currently the case for Southeast Asia (TRAFFIC 2008, Reino et al. 2017, Aloysius et al. 2020). However, concern about the spread of zoonotic diseases or pressure by citizens concerned by the toll of wildlife trade on biodiversity has resulted in many countries adopting trade barriers to partially or totally ban the import or export of wild specimens (table 1). Although such bans can severely suppress the demand for some wildlife products (Reino et al. 2017), their ultimate effectiveness is subject of debate. For example, tigers remain heavily poached to meet demand for traditional medicine for their bones, despite their listing in appendix I of the Convention on International Trade in Endangered Species of Wild Fauna and Flora (CITES, https://cites.org; Stoner and Pervushina 2013). Indeed, measures aimed at restricting the supply of price-inelastic products raise their price but do little to lower demand, potentially exacerbating illegal trade (McNelly 2001).

**Table 1.** Nonexhaustive list of regulations banning the commerce of wild birds worldwide.

Regulation	Year of implementation	Aim	Countries
Convention on International Trade in Endangered Species of Wild Fauna and Flora (CITES)	1975	Ensure that international trade in specimens of wild animals and plants does not threaten their survival.	183 parties
Wild Bird Conservation Act	1992	Ban importations of wild birds Ensure that exotic bird species are not harmed by international trade and encourages wild bird conservation programs in countries of origin. Most bird species listed under the Convention on International Trade of Endangered Species of Wild Fauna and Flora (CITES) are listed under WBCA, exempt: birds native to the 50 states and the District of Columbia; two parrot species: budgie (Melopsittacus undulatus) and cockatiel (Nymphicus hollandicus); birds in families Anatidae, Cracidae, Dromaiinae, Gruidae, Megapodidae, Numididae, Phasianidae, Rheidae, Struthionidae. Wild-captured non-CITES species can still be imported.	United States
Wildlife act	1979	Import ban on wildlife trade	Canada
Wildlife Protection Act (Regulation of Exports and Imports)	1959–1995	Export ban on the trade of any live native animal (1959) Export ban on the trade of parrots (1982) Import: very reduced list of bird species can be legally imported (1995)	Australia
Wildlife act	1953 1997	Export ban on the trade of wildlife Import ban on the trade of wildlife	New Zealand
European Wild Bird Trade Ban	2005	Import ban on the trade wild birds	European Union (27 countries)
Fauna Protection Law	1967	Export ban on the trade wildlife	Brazil
Wildlife, national parks, hunting and fishing law	1984	Export ban on the trade wildlife	Bolivia
National code for natural resources and environmental protection	1978	Export ban on the trade wildlife	Colombia
Law for the protection of wild fauna	1970	Export ban on the trade wildlife	Ecuador

**Box 1. Glossary presenting definitions and relevant concepts used in this manuscript.**

**Demand:** The willingness to purchase a good or service. It can be divided into effective demand (the aggregate amount of transactions made at a given price level), latent demand (the aggregate of transaction not yet made at current market conditions, but for which there is a willingness to purchase if quantities or prices of supply change), and potential demand (sum of effective and latent demand aggregates expected for a given market).

**Demand elasticity:** Responsiveness to changes in price and income. Income-elastic products are those for which demand increases as incomes rise. For example, rising consumer income is a driver of increased demand for wildlife products in Southeast Asia (TRAFFIC 2008). Demand for price-inelastic products is less responsive to price changes, because significant increases in price do little to discourage consumption. For example, tiger bones remain heavily poached to meet demand for traditional medicine, despite their listing in Appendix I of CITES (Stoner and Pervushina 2013).

**Geopolitical (trajectories, dynamics, storylines, scenarios, axes, narratives, factors, or balances):** Geopolitics analyses how political power is affected by geographical arrangements, such as boundaries, coalitions, spatial networks or natural resources. Regarding wildlife trade, increasing restrictions to the trade of wildlife is known to have profound (even if unintentional) effects on the global commerce of wildlife (Reino et al. 2017). On the other hand, foreign assistance supporting interventions addressing the illegal wildlife trade has increased dramatically over the past decade. This “geopolitical ecology of conservation” arose following concerns about threats to national security posed by illicit harvesting and trafficking of wildlife (Massé and Margulies 2020), and can have important effects on invasion and extinction risks worldwide. In our study, we aim to explore how geopolitics might affect wildlife trade focusing on two drivers of trade: the strength of trade barriers and degree of law enforcement.

**Market role:** A country is classified as either mainly supplier or demander of wildlife items. In this study, countries classified as suppliers were those exporting more than what they imported, whereas countries classified as demanders were those countries importing more than they exported. Classifications based on wild bird trade data compiled by CITES (https://trade.cites.org). Although switches between demanding and supplier status can occur over time, we assumed the current status was representative of a given country's market role under the different scenarios considered.

**Potential supply:** Supply that, given item availability, may be provided, but thanks to numerous constraints (e.g., logistic), is not realized.

**Rule of Law:** Country's willingness to enforce laws aimed at market regulation. Regarding this study, the more willing and able a country is to regulate and intervene in its internal market, the stricter may become its conservation stance on wildlife.

**Scenarios:** According to the Intergovernmental Panel on Climate Change (IPCC), a scenario describes a “coherent, internally consistent and plausible description of a possible future state of the world.” Often, as is the case with this study, a set of scenarios is described to capture the range of possible future states of a system (IPCC 2014).

**Storylines:** Define the core of each scenario, describing its main characteristics, drivers and dynamics. Furthermore, they provide information on relationships and feedback loops between key drivers (IPCC 2014).

**Trade barriers:** Trade barriers describe the political willingness to engage in international trade. The stronger the barriers, the more encumbered will be legal commerce between countries. Although this variable measures the political regulation of the markets between countries, it does not necessarily reflect the political regulation of the markets within each country. That is achieved by another variable, representing rule of law, as is defined by those countries’ willingness to enforce laws aimed at market regulation. The more willing and able a country is to regulate and intervene in its internal market, the stricter may become its conservation stance on wildlife trade.

Given the inability of trade bans to solve the poaching crisis for several species (Conrad 2012), direct action against poachers and increased funding of law enforcement in exporting countries have been touted as the most efficient ways to curb excessive collection of wildlife to supply international markets (Holden and Lockyer 2021). Trade agreements can provide strong incentives to improve environmental commitments by both trading partners within their own territories, not only on borders and customs. For example, the European Union's “green deal diplomacy” uses trade “as a platform to engage with trading partners on … environmental action,” focusing on cracking down on illegal wildlife trade and ensuring that non-European countries benefit from biodiversity-friendly trade (European Parliament 2016). Enforcement mechanisms are indeed known to be more efficient in trade agreements than in environmental ones; that is, environmental provisions in regional trade agreements translate into increased environmental provisions in domestic legislation of signatory countries, whereas provisions in international environmental agreements do not (Brandi et al. 2019).

International action can also influence the sourcing of wildlife in more direct ways. For example, foreign assistance supporting interventions to control wildlife trade in exporting countries has increased dramatically over the past decade, with the US being one of the largest providers of assistance to fight wildlife trafficking (Massé and Margulies 2020). Foreign policies are therefore bound to affect wildlife trade, be it through the implementation of regulations motivated by trade dynamics, through the establishment of general trade agreements, or even via direct donations to exporting countries. In fact, because biodiversity conservation and wildlife trade are getting increasingly constrained by the geopolitical contexts, changes to countries’ geopolitics can profoundly affect wildlife trade. Overall and despite its inherent complexity, understanding how geopolitics steer wildlife trade is absolutely pivotal for informing more effective policies and ensuring a globally sustainable commerce of wildlife*.*

## Scenario framework to predict future wildlife trade

Because future political and economic trajectories cannot be forecasted, the alternative is to develop scenarios describing plausible and internally consistent trajectories. Scenarios for the future have been developed and coupled with models to deliver quantitative projections for several aspects of global environmental change such as climate (IPCC 2014), land use (Hurtt et al. 2011), or human population development (Lutz et al. 2014). Scenario building has become crucial for policy- and decision-making, exploring and presenting the potential consequences and impacts of human actions under different future developments (IPBES 2016). Roura-Pascual and colleagues (2021) developed a set of future global biological invasion scenarios implying both commercial and accidental displacement of species, but a link between geopolitical changes and wildlife trade fluxes is still missing.

**Figure 1.**
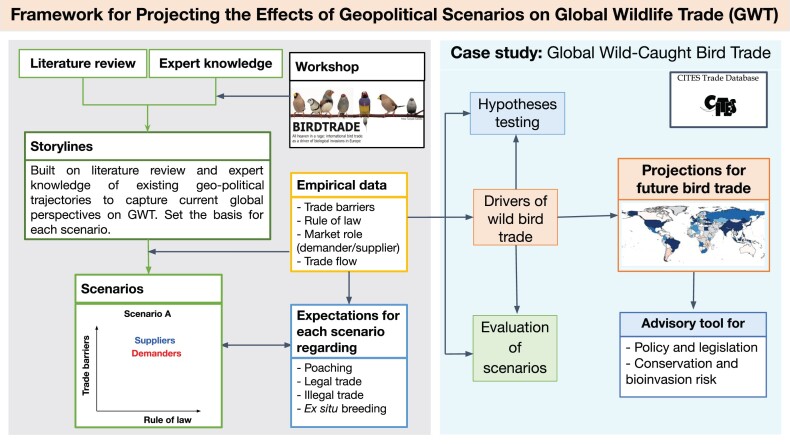
Key steps and elements for developing and applying the presented framework for projecting the effects of geopolitical changes on global wildlife trade. First, the conceptual approach is presented at the left side of the figure, highlighting literature review and expert knowledge as the basis for developing the storylines, from which scenarios were initially conceived. The right side of the figure represents the empirical steps of the framework, including final conceptualization, validation and application using a case study. Data on bird trade, trade barriers and rule of law was collected and used for scenario development, exploration, validation and hypothesis testing, enabling projections for future bird trade in different scenarios (only scenario A is depicted) and providing a valuable tool to guide policy and legislation.

**Figure 2.**
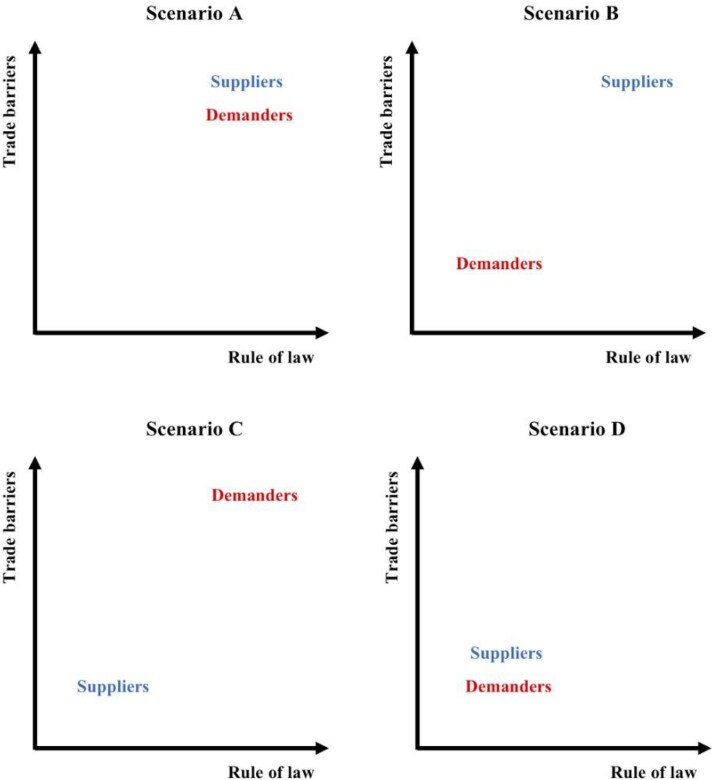
Relative position of suppliers and demanders along the axis rule of law and trade barriers in each of the four scenarios considered. (a) Scenario A: strong trade barriers, strong law enforcement; (b) scenario B: strong trade barriers and law enforcement for suppliers, but weak for demanders; (c) scenario C: strong trade barriers and law enforcement for demanders, but weak for suppliers; (d) scenario D: weak trade barriers, weak law enforcement.

To address this issue, we developed a framework leading to four storyline-based scenarios describing how changes in geopolitics could relate to wildlife trade (figure 1). Having decided beforehand to circumscribe the scope of our analysis to the legal and political drivers of international bird trade, we discussed the implications of loosening (or tightening) bird trade regulations *within* each country—namely, enforcing laws on production, harvesting, and domestic commerce—and the consequences of imposing (or tearing down) trade barriers *between* countries or trade blocs. These two variables, (international) trade barriers and (national) law enforcement are important drivers of bird trade, can be readily characterized by available empirical data and graphically represented in the form of a two axis plane (Van der Heijden 2005). For this study, each axis represents one of the two key drivers of trade (trade barriers and rule of law), and the resulting space may be read according to a 2 × 2 storyline matrix for different driver combinations. Our four orthogonal geopolitical scenarios are therefore based on their policies regarding international trade barriers (i.e., border policies being favorable, with lower costs for import or export versus border policies being unfavorable to trade, with higher costs for import or export) and domestic rule of law (i.e., the effectiveness of law enforcement; box 2, figure 2).

**Figure 3.**
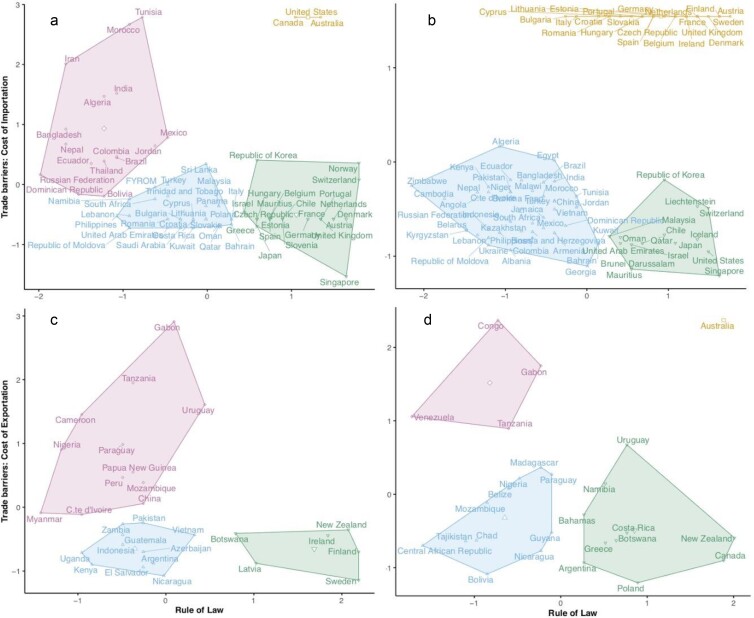
Fuzzy clustering analysis determining where each country would be placed within the four storyline-based scenarios (scenarios A–D). (a) Preban demanders; (b) postban demanders; (c) preban suppliers; (d) postban suppliers. Regarding living wild-caught birds trade, both supplying and demanding countries can be grouped into three (b and c) or four clusters (a and d). Country placement differs given shifts in country's parts as mainly suppliers or demanders of wild-caught live birds or to differences in trade barrier strength, specifically following the EU ban. Colored polygons and text represent cluster membership.

**Box 2. Overview of the storylines drawn from geopolitical axes characterizing the strength of trade barriers and law enforcement.**

**Storyline A: A world of fragmented wealth**

Nationalism and deglobalization are the motto, whereas general economic progress—in spite of being unevenly distributed—enables less prosperous countries to overcome extreme poverty. Most governments become keener on biodiversity conservation as part of state-led dirigiste or Keynesian development plans, even in poorer countries. Wealthier countries embrace economic protectionism and try to decrease as much as possible the volume and composition of their imports—including exotic pets. Environmental and health concerns also motivate more restrictions over international commerce of living animals, to prevent zoonotic pandemics (e.g., SARS-CoV-2). Developing countries respond to deglobalization by improving the effectiveness of their public administration; monetizing their biodiversity through ecotourism and reinforcing their biodiversity conservation authorities, curbing the exportation of wildlife (figures 2 and 3).

**Storyline B: A divided world**

In the likelihood of libertarian laissez faire, laissez passer ideologies taking a hold on the wealthiest countries’ political arena, environmental policies could embrace the new conservation paradigm (Soulé 2014) and economic policies would favor international trade unencumbered by regulations or duties. Developing countries, on the other hand, having become more prosperous thanks to technological windfall gains, also become keener on a more sustainable use of their natural resources and strongly reinforce their natural park and nature conservancy services, imposing very strict recollection quotas on wildlife captures (figures 2 and 3).

**Storyline C: A fractured world**

The wealthiest countries embrace economic and environment protectionism, whereas developing countries are trapped into political anarchy and its accompanying economic and environmental havoc. Among the former, trade barriers on wildlife become very strict, namely through heavy tariffs on imports, not only to prevent money outflow but also to appease environmental activism. Within the latter, wildlife poaching becomes rampant—indeed, uncontrolled (figures 2 and 3).

**Storyline D: A world gone berserk**

Extreme liberalism in prosperous importer countries to be accompanied by extreme deregulation of wildlife recollection in disadvantaged exporter countries. Public opinion in every country relinquishes concerns about biodiversity conservation, adopting a purely utilitarian ethic on environmental issues. Poverty and political disorder in developing countries not only facilitate unregulated trapping of wild animals but also deny the conditions for ex situ breeding to prosper (figures 2 and 3 and table 2).

**Table 2.** Expectations for the scenarios.

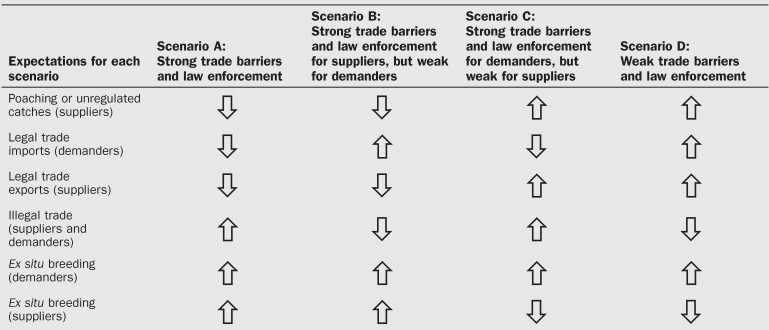

Once the storylines are conceptualized, they can lead to narratives describing plausible trajectories among drivers and actors of wildlife trade. Our approach to storyline development also involves empirical analyses for scenario and hypothesis testing to evaluate storyline consistency, identify additional drivers, and ultimately obtain quantitative projections for future wildlife trade.

### Building scenarios for future trajectories of wildlife trade

Scenarios were developed in a participatory process. During a first workshop organized in the context of project BIRDTRADE's kickoff meeting (Évora, 29 April 2017), team members discussed and outlined the first general ideas about this study. Then, on 29 May 2017, a smaller meeting took place, during which the authors outlined the first tentative scenarios. A total of 20 experts from Portugal, Spain, Argentina, the United States, South Africa, Italy, and Belgium attended the workshops, representing expertise in invasion science, ecology, global change biology, environmental economy, and policy management.

The outcome was the first set of geopolitical scenarios with measurable consequences for wildlife trade (box 2). The scenarios are nonexhaustive and represent polarized outcomes expected for legal trade, but they provide a set of what-if narratives linking geopolitics and wildlife trade. Although these scenarios outline different possible paths for global trade and its regulations, in the present article, we simplify them along two geopolitical axes characterizing the strength of international trade barriers and the degree of domestic law enforcement. This simplification allows us to assess real-word scenario validity using empirical data on country-specific socioeconomic and bird trade data. Possible outcomes of the alternative scenarios on wildlife trade include increased or decreased poaching, illegal trade fluxes and *ex situ* breeding by both supplier and demand countries (box 2).

### Case study

We used the trade of live wild-caught birds as a case study to test the effects of trade barriers on wildlife trade and validate our approach (figure 1). We relied on the CITES Trade Database (https://trade.cites.org), managed by the UNEP World Conservation Monitoring Centre. It records trade between 183 signatory countries, holding over 18 million records of trade in CITES-listed wildlife. For birds, which are among the most heavily traded taxa worldwide (Reino et al. 2017), the CITES appendices cover about 1700 out of around 2600 bird species known to be traded internationally (approximately 65%, Reino et al. 2017). CITES trade data are often used as broadly representative snapshots of the global legal trade in wildlife (Can et al. 2019) and cover some of the most invasive bird genera, such as nearly all of Psittaciformes, while capturing most of the trade volumes of Passeriformes as well (Cardador et al. 2017, Reino et al. 2017). However, the CITES trade database relies on information communicated by governments, which are not free of errors or biases. For example, governments might occasionally fail to correctly report transactions, species might be misidentified or traded amounts poorly estimated (Reino et al. 2017). Despite these caveats, CITES represents the only global legally binding convention addressing international wildlife trade in a structured and verifiable manner, constituting a valuable source of information to assess the relationships between conservation risks and international trade (Phelps et al. 2010, Hierink et al. 2020). Therefore, we use available data reporting wild-caught specimens from every bird species listed in CITES and legally traded worldwide, in conjunction with the implementation of a ban on the import of living wild birds into the European Union (Cardador et al. 2017), as an opportunity to empirically test the effect of trade barriers on wildlife trade and to validate our approach. Considering the effectiveness of the 2005 EU trade ban on wild birds (Cardador et al. 2017), we compiled data for two time periods: before (1995–2005) and after (2006–2017) the ban. This abrupt, unilateral implementation of a barrier to the import of wild-caught birds is particularly suited to test the accuracy of our framework, because it has been shown to cause major shifts in trade fluxes (Reino et al. 2017).

### Evaluation of scenarios

To examine the internal consistency of our scenarios, we clustered countries based on empirical data on international trade barriers and domestic law enforcement. Along these two axes we examined whether current empirical fluxes of trade matched those expected according to our scenarios (e.g., whether suppliers with low trade barriers and law enforcement would export more live wild birds than those with high trade barriers and law enforcement, see box 2).

The procedure started with the classification of each country as either mainly importer or exporter in the bird trade (suppliers and demanders, respectively), based on CITES data (see the supplemental material for more information).

Then, we plotted supply and demand countries, independently, in the two-axes fuzzy ordination diagram defining strength of trade barriers (cost of exportation or import tariffs; supplemental table S1) and law enforcement (rule of law; supplemental table S1, figure 3), before and after the EU ban on bird trade (see the supplemental material for further details). Fuzzy *c*-means clustering (Bezdek 1974) was used to visualize how well each country fits within each one of the four scenarios proposed (e.g., demanding countries with strong trade barriers and rule of law or supplying countries with strong trade barriers but low rule of law; see figure 3, box 2). The analysis revealed that both supply and demand countries can be grouped into three (figure 3c) or four well-defined clusters (figure 3c, 3b, and 3d), representing different combinations of trade barrier strength and degree of rule of law (see the supplemental material for more information).

**Figure 4.**
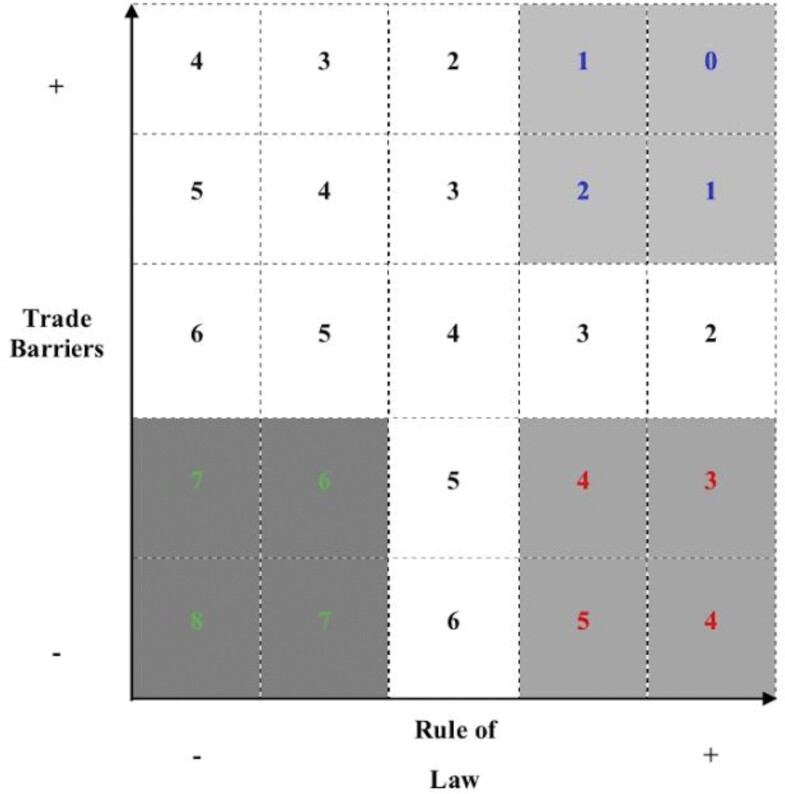
Scores devised to translate the expected importance of a given country as supplier or demander, depending on its relative position in a plot considering trade barriers and rule of law. According to this score, a supplying country with low scores for rule of law and trade barriers (cost of exportation) is expected to be an important exporter, and is therefore attributed a high expected score, in this case, a score of 6 to 8 (bottom left corner, dark gray background, green numbers). On the other hand, a demand country (mainly importer) with high rule of law and low trade barriers (import tariffs) is expected to be a relevant importer and is therefore attributed a low expected score, in this case, a score of 3 to 5 (bottom right corner, lighter gray background, red numbers).

Then, we measured how well each country's geopolitical profile (i.e., position within the two-axes diagram defined by trade barriers and rule of law) explains the number of traded birds between supply and demand countries, before and after the EU ban. More specifically, we devised two scores. The first, represents the expected relative importance of each country as a demander or supplier of wild birds based on the country's position within the two-dimensional coordinate system defined by trade barriers strength and degree of rule of law (expected score). The second, represents the importance of each country as a demander or a supplier of wild birds, based on the amount of live wild birds traded by each country (e.g., the biggest suppliers had the highest scores; real trade score). For the expected score, we binned trade barriers and rule of law data for each country, so that it ranged from 0 (strong rule of law and trade barriers) to 8 (weak rule of law and trade barriers; figure 4). For the real trade score, we log-transformed and binned the amount of wild live birds exported and imported by every country to correct the high skewness of the real trade data. The real trade score therefore ranged from 1 (countries with low importance in bird trade) to 5 (heavy traders of live wild birds). We then compared the two scores and tested whether a given country's trade barriers and rule of law (expected score) can predict its importance as a demander or a supplier of wild birds (real trade score). To test whether, for example, higher expected scores are characteristic of heavy suppliers or lower expected scores of big demanders, we fit an ordinal regression (R package clmm; Christensen 2011) between the expected scores and the real trade scores for each country (suppliers and demanders).

**Figure 5.**
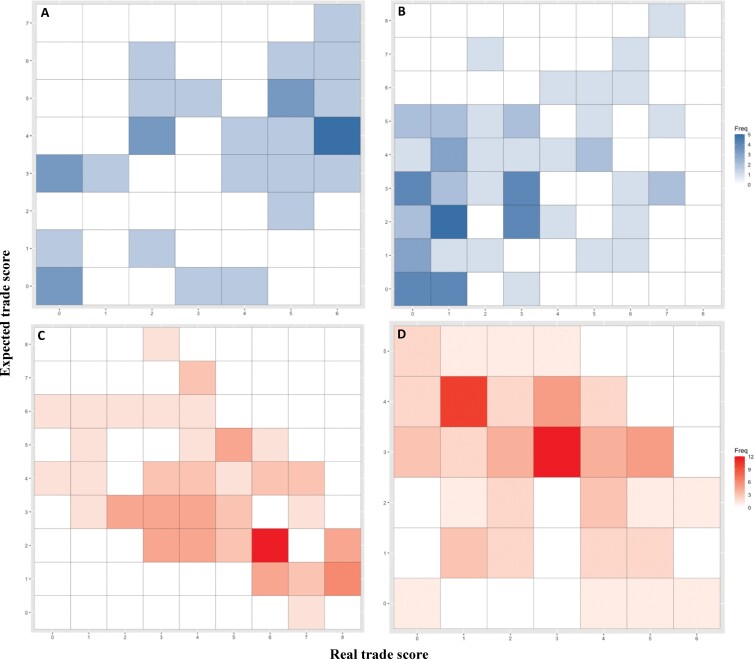
Heatmaps for each country's expected scores plotted against respective real trade score calculated using exported and imported amounts—that is, a plot of the binned representation of the actual trade for both supplying and demanding countries as a function of the score devised to predict the amount of trade. The color intensity represents the frequency with which each country's expected score matches its observed trade score. Given the high skewness of the raw data, we log-transformed and binned the actual amount of wild live birds exported and imported by every country, using equal intervals. The countries with low importance in bird trade were binned as 1, and heavy traders were binned as 5. The expected scores for suppliers were positively associated with real trade scores, whereas demanders’ expected scores were negatively associated with the respective real trade scores (a) Preban suppliers (β = .500, SE = 0.189, p = .008, Nagelkerke's R^2^ = .24, p < .01); (b) postban suppliers (β = .400, SE = 0.123, p = .001, Nagelkerke's R^2^ = .16, p < .01); (c) preban demanders (β = –.575, SE = 0.136, p < .01, Nagelkerke's R^2^ = .26, p < .01); (d) postban demanders (β = –.578, SE = 0.198, p = .003, Nagelkerke's R^2^ = .26, p < .01).

Expected scores for suppliers were positively associated with real trade scores (preban, *β* = .500, standard error [SE] = 0.189, *p* ≤ .01, Nagelkerke's *R*^2^ = .24, *p* < .01; postban, *β* = .400, SE = 0.123, *p* < .01, Nagelkerke's *R*^2^ = .16, *p* < .01), whereas demanders’ expected scores were negatively associated with the respective real trade scores, both before and after the EU ban (preban, *β* = –.575, SE = 0.136, *p* < .01, Nagelkerke's *R*^2^ = .26, *p* < .01; postban, *β* = –.578, SE = 0.198, *p* < .01, Nagelkerke's *R*^2^ = .26, *p* < .01; figure 5). According to McFadden (1977), pseudo *R*^2^ values of .2–.4 represent an excellent model fit. Therefore, our results suggest that the expected score adequately represents the actual amount of wild birds traded by a given country, being positively associated with the amount of birds supplied and inversely associated with the amount of birds demanded. These results are consistent with the wildlife-trade outcomes associated with the alternative storyline-based scenarios developed, therefore supporting the broader use of our scenarios as exploratory tools for policy assessment and decision-making.

### Projections of trade volumes in different scenarios

Using the ordinal regression models fit between expected and real trade scores of suppliers and demanders following the EU ban, we obtained estimates of the amount of live wild birds traded in different scenarios. For example, in scenario A, both suppliers and demanders experience high trade barriers and rule of law, therefore being represented by an expected score between 0 and 2 (figure 4, upper left corner). We randomly attributed a score within that range to each supplier and demander country, and used the calibrated ordinal regression models to obtain projections for their real trade scores, based on the expected trade scores randomly attributed within the appropriate range for scenario A.

**Figure 6.**
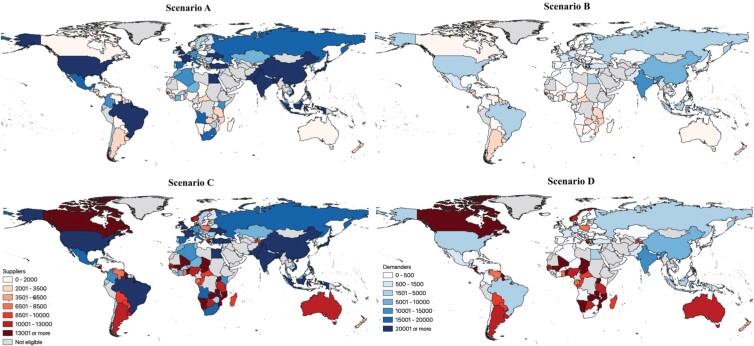
World maps showing projected amounts of live wild caught birds supplied and demanded for all countries considered for this analysis, and each scenario. Scenario A: strong trade barriers, and rule of law; protectionist demanders and suppliers. Scenario B: strong trade barriers and law enforcement for suppliers, but weak for demanders; libertarian demanders and regulated suppliers. Scenario C: strong trade barriers and law enforcement for demanders, but weak for suppliers; protectionist demanders and libertarian suppliers. Scenario D: weak trade barriers, weak rule of law; libertarian demanders and suppliers. See supplemental figures S5 and S6 for more information on projected amounts for suppliers and demanders in each scenario.

Our projections suggest the volumes of living wild birds traded globally will increase under scenario C (i.e., protectionist demanders and libertarian suppliers; strong trade barriers and weak rule of law, box 2; figure 6 and supplemental figure S6). Despite protectionism from developed demanders of live wild birds, who impose heavy tariffs on wildlife imports, wildlife suppliers face political turmoil and socioeconomic crisis with the consequent increases in poaching and illegal exportation. Although it is exaggerated, this is the most palpable scenario considering the current global reality. Increasing regulations on imports may therefore not cause the desired decrease in exports, as long as there is market demand and suppliers are heavily reliant on exports (Ferreira and Okita-Ouma 2012). Accordingly, our projections indicate that the amount of live wild birds will decrease under scenario B (i.e., libertarian demanders face regulated suppliers; box 2, figures 6 and S6). In this case, developed demanders would promote free international trade of live wild birds, but more prosperous supplier countries would promote a more sustainable use of their natural resources, imposing very strict collection quotas on wildlife. Overall and contrary to what could be expected, our projections indicate scenarios A and C (both with high trade barriers and rule of law demanders) will express strong demand, whereas scenarios D and B (both with weak trade barriers and rule of law suppliers) are expected to experience higher supply of wild birds globally, even superior to demand (figures 6 and S6).

## Wildlife trade across different scenarios of future change

This study shows the complex ecological and socioeconomic factors associated with the global wildlife crisis. Consideration of factors related to social stability and trade development in global scenarios and models is therefore essential to adequately anticipate environmental risks posed by wildlife trade. Our study proposes a new conceptual approach for the development of geopolitical scenarios relevant to wildlife trade and for exploring their expected consequences on the direction and magnitude of trade fluxes between supply and demand countries. The proposed approach builds on the development of storyline-based scenarios using two axes: the strength of international trade barriers and degree of domestic rule of law. We examined how scenarios help discriminate between categories of supply and demand countries and demonstrate that they predict reasonably well the historical trends in the global trade of live CITES-listed wild-caught birds. We recognize that CITES trade data limitations might hamper broader generalization of our results to other wildlife trade contexts. Nonetheless, CITES trade records have been reliable surrogates for international wildlife trade fluxes in over 144 peer-reviewed publications, providing an unparalleled tool for monitoring trade in wildlife across borders and understanding global wildlife trade (Robinson and Sinovas 2018). Data availability, bird popularity in wildlife trade, and the recent imposition of a trade ban on wild-caught birds made wild bird trade the ideal case study for our framework. Our results suggest that, depending on which scenario the world's geopolitical balances move into, each country may shift its role in the international bird trade, either changing the quantities it trades or the type of product it trades (wild caught or captivity bred). Such changes are likely to alter global extinction and invasion risks. Forecasting future geopolitical dynamics is difficult, but our results lend support for the use of scenarios as a strategy to explore possible pathways in wildlife trade.

Our scenarios suggest that a future reinforcement of protectionist measures by demanding countries might be counterproductive, leading to an increase in the demand of wild-caught animals and a boost to the illegal trade (Ribeiro et al. 2020). Maximum demand could occur in scenarios with high trade barriers on demanders (A and C), regardless of the situation in supplying countries. If strict rule of law and trade barriers prevail on the demand side, birds may start to be seen as scarce and more expensive, (paradoxically) increasing its demand. Scarcity of a collectable item is known to increase its value and stimulate its demand among collectors (Courchamp et al. 2006). For example, buyers of caged birds value species rarity, mostly defined by its trade availability. Supply for rare species is inelastic, with market arrivals being insensitive to price changes, probably because of a declining stock of rare species in the wild (Krishna et al. 2019). In fact, Krishna and colleagues (2019) and Hanley and colleagues (2018) found that provision of information on rarity actually increased consumer demand. Other studies warn that increased protection and regulation—including CITES listing—may unintentionally cause an increase in value of some species among collectors (Guttery et al. 2016, Janssen and Krishnasamy 2018). After Brazil became the first country in South America to legally ban the commercial sale of wild animals in 1967, thousands of birds were captured to supply international trade, many of them laundered through countries where exports were still legal (i.e., Argentina, Bolivia, and Paraguay; Ortiz-von Halle 2018). Demand for these price-inelastic products is usually unresponsive to trade barriers or price changes, because significant increases in price do little to discourage consumption. In this case, restricting supply through trade restrictions or quotas will raise the price but do little to lower demand. As such, price increases may exacerbate illegal trade to meet demand (McNelly 2001). For example, rhino horns remain heavily poached to meet demand, despite trade bans (Eikelboom et al. 2020). Sadovy de Mitcheson and colleagues (2018) made a case for banning altogether the demand for wildlife products such as shark fin, because simply constricting the trade increases their market value and therefore creates an incentive to poaching. Likewise, Heltberg (2001) posited that the CITES ivory trade ban would only reduce poaching if, among other conditions, it has a large demand-reducing effect. Despite controversial, blanket bans have been shown to curb trade; following the EU ban on the importation of wild live birds, fluxes of global bird trade declined sharply (Reino et al. 2017). Nonetheless, although regional bans can decrease invasion risk globally, to be fully effective and prevent rerouting trade flows, bans should be global (Reino et al. 2017) and—as our results suggest—implemented along with demand-reducing campaigns and policies.

Contrary to initial expectations, our projections reveal that the demand for live CITES-listed wild-caught birds is low within a scenario of weak trade barriers (B and D). Notably, in these situations import volume diminishes while the potential stock for exportation expands, which may cause prices for traded items to decrease (Ferreira and Okita-Ouma 2012). This means that when birds’ availability to consumers in demand countries ceases to be constrained, birds might stop being considered rare and valuable items, the willingness to pay diminishes as the supply becomes plentiful, and the volume traded may even decrease, eventually setting itself on a modest level. Besides, in such a *laissez faire* context, local breeding of exotic species becomes legal and may provide additional supply. This may have serious consequences for both native populations exploited in supplying regions and invasive alien species emerging in importing parties. Although legal import volume is not high, the high availability and supply of wild-caught specimens may result in an increase in illegal trade, ultimately generating considerable propagule pressure in receiving regions—a decisive driver of invasive alien species emergence (Simberloff 2009, Cassey et al. 2018)—which, coupled with the items’ low price, may increase the probability of accidental and intentional escapes.

## Conclusions

The global environmental crisis pleads for effective internationally coordinated policies, but changes to the global sociopolitical order may compromise such efforts. Like many other economic activities, wildlife trade is regulated by political and economic dynamics, and shifting fluxes of international trade can have knock-on effects on the fluxes of traded birds themselves (Reino et al. 2017). Because predictions of future geopolitical trajectories is impossible, a deductive approach for building scenarios, coupled with a systematic assessment of how key drivers can alter future trends is critical to support policy assessment and decision-making. As far as we are aware, we provide the first geopolitical narratives designed to infer global wildlife trade dynamics and assess their consequences for wildlife trade fluxes between supply and demand countries. Although further testing and refining will be welcomed, we show that the expectations associated with the narratives developed match empirical patterns of wildlife trade. It may be useful for helping build and increase awareness of the necessity of considering geopolitics and socioeconomics into conservation and biological invasion models, practices, and legislation. Increasing restrictions risks fueling an inscrutable, uncontrolled, and highly priced illegal trade sustained by rising incomes and social status of growing middle classes (Ribeiro et al. 2020). Our main recommendations for reducing the negative impacts of wildlife trade on conservation and biological invasions are for policymakers to work toward improving domestic and international law enforcement, because poorly policed trade controls can allow illegal trade to flourish inscrutably; toward widely implementing and prioritizing initiatives to dissuade wildlife consumption in demanding countries, such as well-organized and wisely directed education campaigns (Margulies et al. 2019, Ribeiro et al. 2020) to discourage the purchase of wildlife, satisfying demand with controlled captive-bred individuals posing lower conservation risks (Carrete and Tella, 2008, 2015); and toward addressing inequalities between trading states, possibly through incentive or compensation-driven programs similar to other international environmental initiatives, such as REDD+ (Liew et al. 2021). Ultimately, if legal restrictions are to be imposed on trade, they must be implemented at the global scale.

## Supplementary Material

biac015_Supplemental_Files

## Notes

Joana Ribeiro (joanatribeiro@cibio.up.pt) and Joana Santana (joanafsantana@cibio.up.pt) are postdoc researchers and Luís Reino (luis.reino@cibio.up.pt) is an ecologist and ornithologist at CIBIO/InBIO, BIOPOLIS, at the University of Porto, in Porto, Portugal. Pedro Bingre (bingre@esac.pt) is a forester and land use planner lecturing at Coimbra Polytechnic, in Coimbra, Portugal. Diederik Strubbe (diederik.strubbe@ugent.be) is a biogeographer and ecologist from the University of Ghent, in Ghent, Belgium. César Capinha (cesarcapinha@campus.ul.pt) is a researcher at the Centre for Geographical Studies of the University of Lisbon, in Lisbon, Portugal. Miguel B. Araújo (maraujo@mncn.csic.es) is a research professor at the MNCN-CSIC, in Madrid, Spain, and a professor and chair of biodiversity at the University of Évora, in Évora, Portugal.
