# Supplementary material for: Exploring the Effects of Geopolitical Shifts on Global Wildlife Trade
Source: Bioscience. 2022 Apr 6;72(6):560–72. doi: 10.1093/biosci/biac015 (PMC9180917; doi:10.1093/biosci/biac015)
Supplement: biac015_Supplemental_Files [file biac015_supplemental_files.zip › Supplementary_Material.docx]

**Supplementary material for:**

**Exploring the effects of geo-political shifts on global wildlife trade**

Joana Ribeiro, Pedro Bingre, Diederik Strubbe, Joana Santana, César Capinha, Miguel B. Araújo, Luís Reino

**Table S1.** Socioeconomic and environmental variables used to describe the conceptualized scenarios.

| **Variables** | **Definition** | **Rationale** | **Source** |
| --- | --- | --- | --- |
| **CITES bird species** | Number of bird species listed in CITES annexes, for each country. Variable used for supplying countries only. | Relevant for supplying countries, to describe the availability of wildlife products to export. | http://checklist.cites.org |
| **CO_2_ emissions** | Carbon dioxide emissions are those stemming from the burning of fossil fuels and the manufacture of cement. They include carbon dioxide produced during consumption of solid, liquid, and gas fuels and gas flaring. | Trade openness and interaction between economic growth and renewable electricity consumption exert a positive impact on CO2 emissions (Balsalobre-Lorente et al. 2018). | https://data.worldbank.org |
| **Colonial past (Colonial past/No colonial past)** | Binary variable, describing whether a country had a colonial past or not; i.e. if I colonized other countries. | Countries with a colonialwith colonial past typically have close trading relations with former colonies. Particularly relevant for demanding countries. |  |
| **Control of corruption (EIU17CC)** | Corruption among public officials | Proxy for rule of law. | Economist Intelligence Unit  https://www.eiu.com |
| **Corruption perception index (CPI)** | The index, which ranks 180 countries and territories by their perceived levels of public sector corruption according to experts and businesspeople, uses a scale of 0 to 100, where 0 is highly corrupt and 100 is very clean. | Corruption is a clear facilitator of the illegal trade in wildlife (Wyatt et al. 2018). | https://www.transparency.org |
| **Electric power consumption** | Electric power consumption measures the production of power plants and combined heat and power plants less transmission, distribution, and transformation losses and own use by heat and power plants. | Proxy to GDPpc | https://data.worldbank.org |
| **Energy use** | Energy use refers to use of primary energy before transformation to other end-use fuels, which is equal to indigenous production plus imports and stock changes, minus exports and fuels supplied to ships and aircraft engaged in international transport. | Increased per capita energy use is commonly associated with increased economic growth (Stern 2011), and may be linked with increased income available to spend on superfluous items, such as exotic pets. Particularly useful for demanding countries. | https://data.worldbank.org |
| **Environmental performance index (EPI)** | The 2018 Environmental Performance Index (EPI) ranks 180 countries on 24 performance indicators across ten issue categories covering environmental health and ecosystem vitality. | These metrics provide a gauge at a national scale of how close countries are to established environmental policy goals. | Wendling et al. (2018) <https://epi.yale.edu/> |
| **Gini index**  **(GINI)** | Gini index measures the extent to which the distribution of income (or, in some cases, consumption expenditure) among individuals or households within an economy deviates from a perfectly equal distribution. A Gini index of 0 represents perfect equality, while an index of 100 implies perfect inequality. | Greater participation in trade significantly reduces income inequality (Chakrabarti 2000). For supplying countries, increased Gini may represent decreased need to resort to overexploiting wildlife for trade, while for demanding countries, might describe an increased economic availability to buy superfluous items, like exotic pets. | https://data.worldbank.org |
| **Government effectiveness (EIU17GE)** | Composite indicator accounting for quality of bureaucracy/institutional effectiveness and excessive bureaucracy/red tape | Proxy for rule of law. | Economist Intelligence Unit  https://www.eiu.com |
| **Gross domestic product per capita (GDPpc)** | GDP per capita is gross domestic product divided by midyear population. GDP at purchaser's prices is the sum of gross value added by all resident producers in the economy plus any product taxes and minus any subsidies not included in the value of the products. It is calculated without making deductions for depreciation of fabricated assets or for depletion and degradation of natural resources. | Proxy for the amount of income available to spend on superfluous items, such as exotic pets. Particularly useful for demanding countries. | https://data.worldbank.org |
| **Inflation** | Inflation as measured by the consumer price index reflects the annual percentage change in the cost to the average consumer of acquiring a basket of goods and services that may be fixed or changed at specified intervals, such as yearly. The Laspeyres formula is generally used. | In supplying countries, inflation leads to a decline in exports because of an increase in product and services’ cost. In demanding countries, due to inflation, money supply enhances in the marketplace, further boosting the purchasing power of people and incrementing the demand for goods and services. In order to fulfill all these demands, the government starts importing products and services. | https://data.worldbank.org |
| **Political stability and absence of violence (EIU17PV)** | Composite indicator accounting for orderly transfers, armed conflict, violent demonstrations, social unrest, international tensions/terrorist threat | Proxy for rule of law. | Economist Intelligence Unit  https://www.eiu.com |
| **Population density** | Human population density is midyear population divided by land area in square kilometers. Population is based on the de facto definition of population, which counts all residents regardless of legal status or citizenship-except for refugees not permanently settled in the country of asylum, who are generally considered part of the population of their country of origin. Land area is a country's total area, excluding area under inland water bodies, national claims to continental shelf, and exclusive economic zones. In most cases the definition of inland water bodies includes major rivers and lakes. | Increasing human population density has been associated with enhanced both consumptive and non-consumptive forms of wildlife-based economic activities (Masanja 2014). In demanding countries, human population density explains alien species richness (Spear et al. 2013). | https://data.worldbank.org |
| **Regulatory Quality**  **(EIU17RQ)** | Composite indicator including: Unfair competitive practices; Price controls; Discriminatory tariffs; Excessive protections; Discriminatory taxes | Proxy for rule of law and trade barriers. | Economist Intelligence Unit  https://www.eiu.com |
| **Rule of law** | Composite indicator accounting for violent crime, organized crime, fairness of judicial process, enforceability of contracts, speediness of judicial process, confiscation/expropriation, intellectual property rights protection and private property protection. | One of the two aspects used to empirically test our approach and the validity of our scenarios. | Economist Intelligence Unit  https://www.eiu.com |
| **Trade barriers: Cost of exportation** | Border compliance captures the time and cost associated with compliance with the economy’s customs regulations and with regulations relating to other inspections that are mandatory in order for the shipment to cross the economy’s border, as well as the time and cost for handling that takes place at its port or border. The time and cost for this segment include time and cost for customs clearance and inspection procedures conducted by other government agencies. | Proxy to trade barrier to supplying countries. | https://data.worldbank.org |
| **Trade barriers: Import tariffs** | Tariffs imposed by all countries on imported items. | Proxy to trade barrier to demanding countries. | http://tariffdata.wto.org |
| **Unemployment** | Unemployment refers to the share of the labor force that is without work but available for and seeking employment. Definitions of labor force and unemployment differ by country. | Increased unemployment has been associated with increased poaching (Ngwakwe and Mokgalong 2014). Particularly relevant for supplying countries. | https://data.worldbank.org |
| **Urban population** | Urban population refers to people living in urban areas as defined by national statistical offices. The data are collected and smoothed by the United Nations Population Division. | Proxy to GDPpc | https://data.worldbank.org |
| **Voice and accountability (EIU17VA)** | Composite indicator including: Democracy Index; Vested interests; Accountability of Public Officials; Human Rights; Freedom of association | Proxy for rule of law. | Economist Intelligence Unit  https://www.eiu.com |

**Supplementary Methods**

**Data selection**

We queried the CITES trade database to retrieve data on wild-caught live avian specimens from every listed bird species legally traded among all countries from 1995 to 2005 (pre-EU ban period) and from 2006 to 2017 (post-EU ban period), for all purposes. We focused on birds reported as wild-caught because its capture from the wild is a major threat to native populations, and also associated to increased potential for invasion (Carrete & Tella 2008). Afterwards, each country’s records for exportations and importations were summed, and according to amount exported and imported, each country was categorized as either mainly a supplier or demander of wild-caught live birds before and after the EU ban. Accordingly, some countries had roughly similar amounts of exportations and importations, and were excluded from following analyses, as their part in the trade of live wild-caught birds could not be satisfactorily categorized as either mainly suppliers or demanders. We assumed this simplification of global trade fluxes, as it allowed us to validate our scenarios whilst not imposing significant limitations to our conclusions, i.e., we considered a total of 118 countries as either mainly supplying or demanding live wild birds, whereas only 64 countries behaved as both suppliers and demanders of live wild birds.

**Visualizing each country’s placement within each scenario**

We performed fuzzy clustering (Bezdek, 1974) using each country’s strength of trade barriers (Cost of exportation/Import tariffs, Table S1) and degree of rule of law (Rule of law, Table S1), to visualize where each country would be placed within the four storyline-based scenarios (scenarios A to D, Fig. 2). For instance, in scenario *x,* would a given country be among the group of demander countries with weak rule of law and strong trade barriers, or some other group?

Fuzzy clustering provides an assessment of group membership that accounts for uncertainty in the classification. Countries clearly associated with a given class are assigned high values to that class, while countries with characteristics pertaining to different classes will have intermediate membership values shared among classes. We performed fuzzy c-means clustering using R statistical software (Team and R Development Core Team 2017), with function ‘fcm’ from package ppclust (Cebeci et al. 2019).

To improve scenario description, several other socioeconomic or environmental variables (**Table S1**) were added as descriptors of each country’s socio-economic reality (e.g., a colonial past, or a low environmental performance index). We performed non-metric multidimensional scaling (NMDS) on the fuzzy membership degrees of each supplying and demanding country. We performed NMDS using Bray-Curtis dissimilarity index using function ‘metaMDS’ from R package ‘vegan’ (Oksanen 2015). Following NMDS, we fitted socioeconomic or environmental vectors/factors onto the ordination to identify additional variables correlated with each scenario, using function ‘envfit’ from R package vegan (Oksanen 2015).

**Supplementary Results**

**Results on scenario-building**

Fuzzy clustering analysis revealed that both supplying and demanding countries could be grouped into three (**Fig. 3C**) or four clusters (**Fig. 3A, B** and **D**). NMDS (stress_suppliers_ = 0.019; stress_demanders_=0.028) identified additional variables (see Table S1) associated with trade barriers and rule of law (**Fig. S1-4**, Supplementary Material). Before the EU ban, suppliers with average strength of the rule of law but with high costs of exportation also had high Gini index and traded a high number of bird species listed in CITES annexes, while suppliers with low costs of exportations but a strong rule of law were characterized by higher human population densities (**Fig. S1**). Demanding countries with high trade barriers and strong rule of law also had high GDP per capita or Consumer Price Index (CPI), while countries with high trade barriers but a weak rule of law had high unemployment (**Fig. S2**). Following the EU ban, suppliers with high costs of exportation and strong rule of law have high GDP per capita and CPI (**Fig. S3**), while demanders with low trade barriers and a weak rule of law also have higher Gini index values (**Fig. S4**).


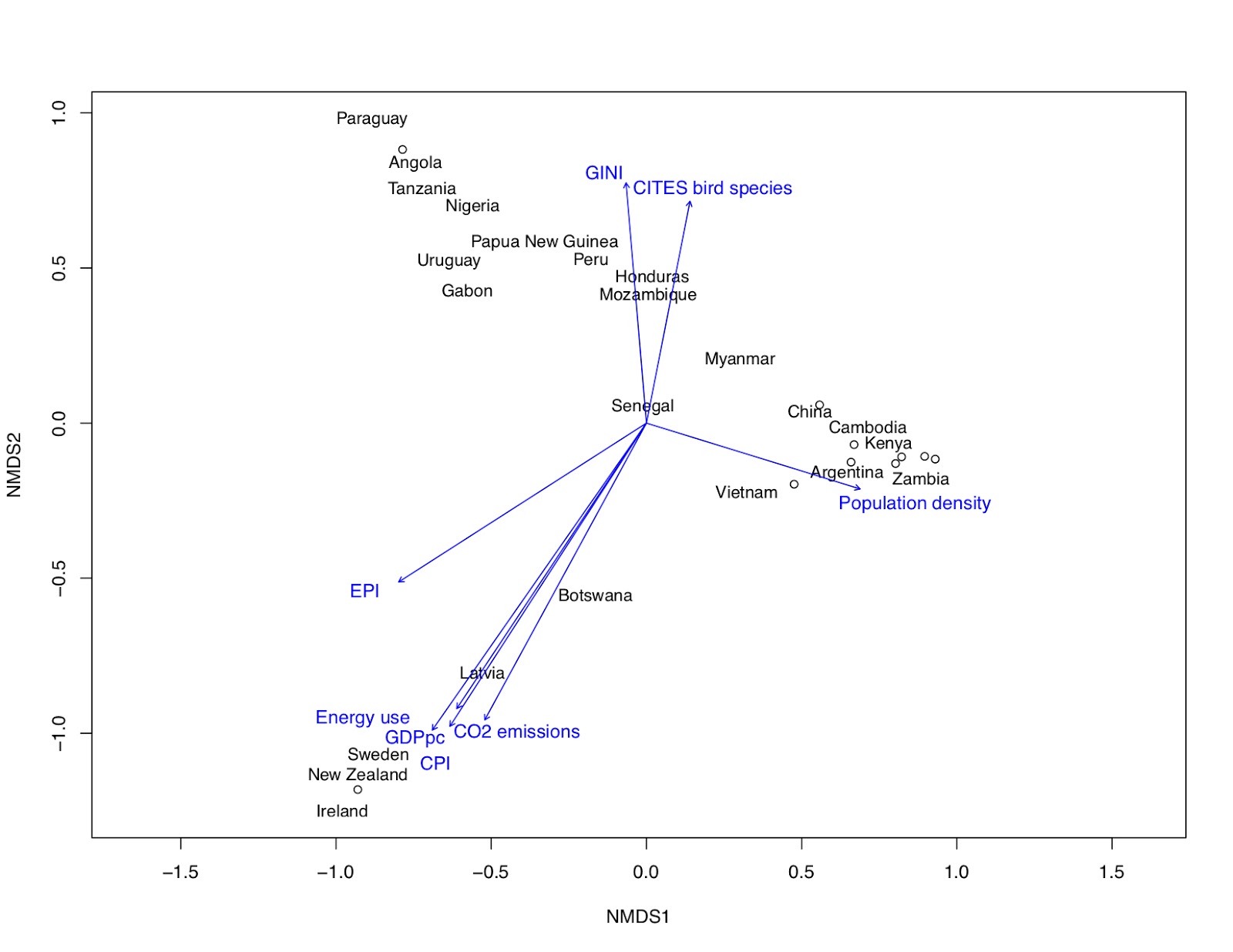


**Figure S1.** Non-metric multidimensional scaling of wild bird suppliers, from 1995-2005 (before the EU ban) with additional predictors correlated with the fuzzy clustering of countries in each scenario.

*
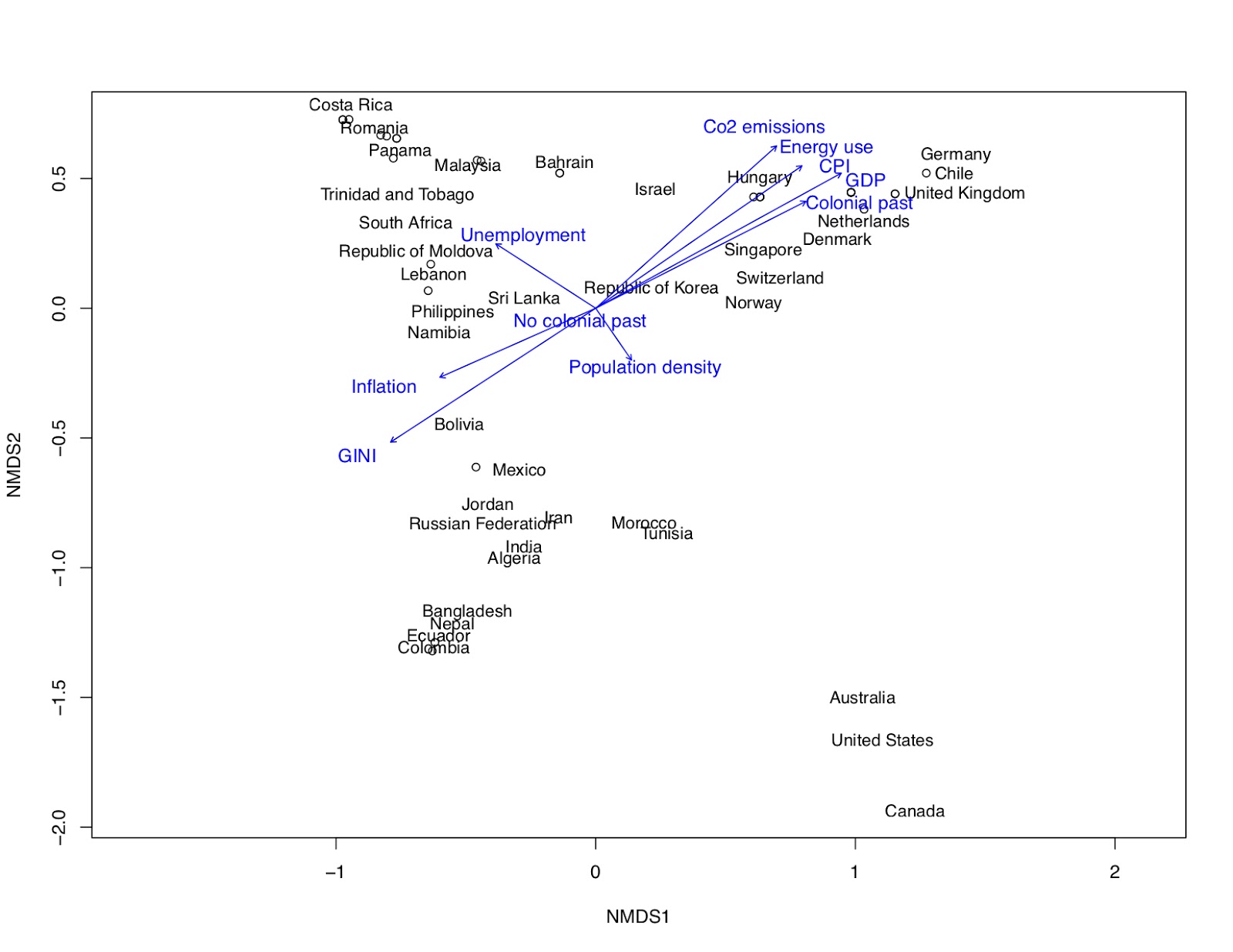
*

**Figure S2.** Non-metric multidimensional scaling of wild bird demanders, from 1995-2005 (before the EU ban) with additional predictors correlated with the fuzzy clustering of countries in each scenario.


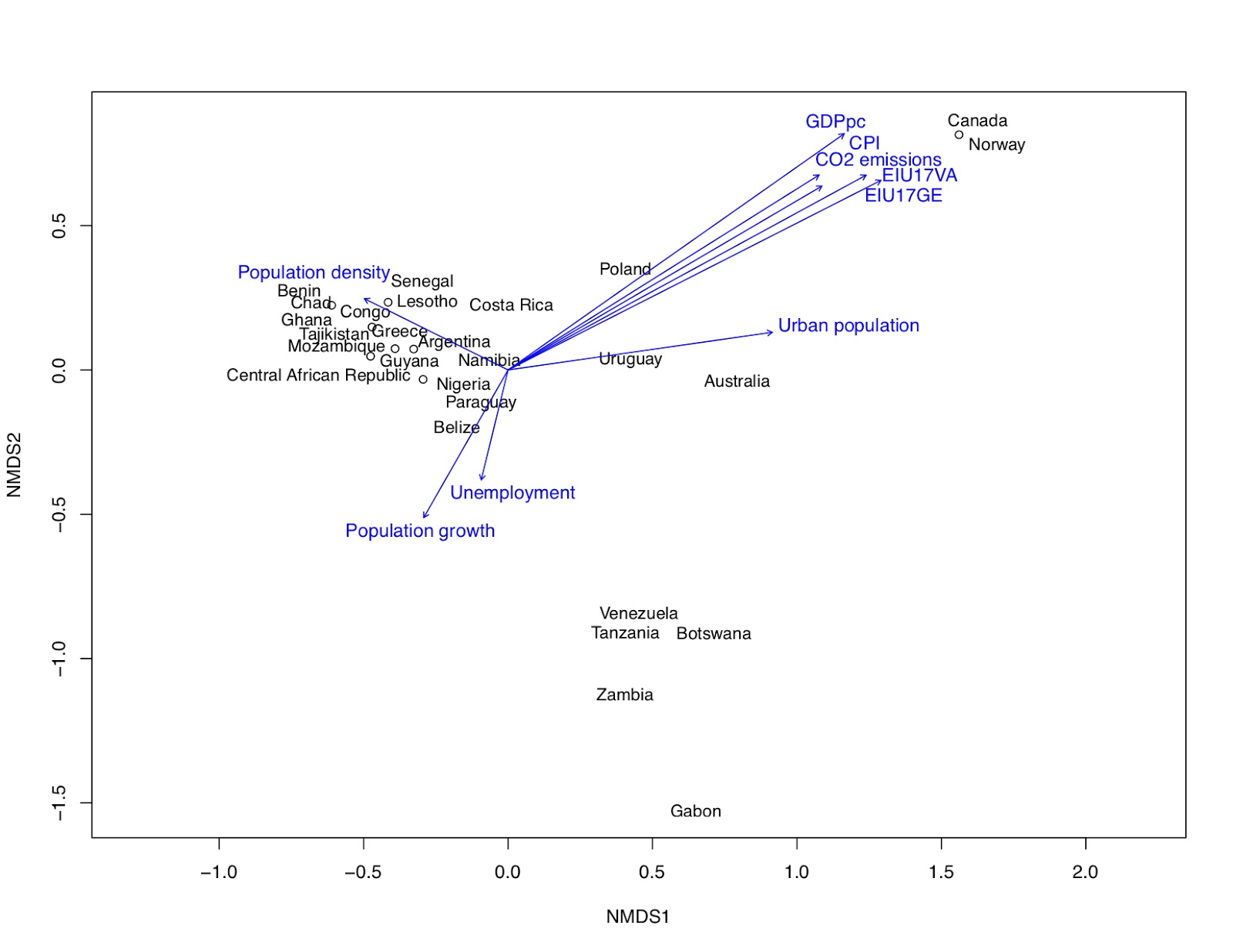


**Figure S3.** Non-metric multidimensional scaling of wild bird suppliers, from 2006-2017 (after the EU ban) with additional predictors correlated with the fuzzy clustering of countries in each scenario.


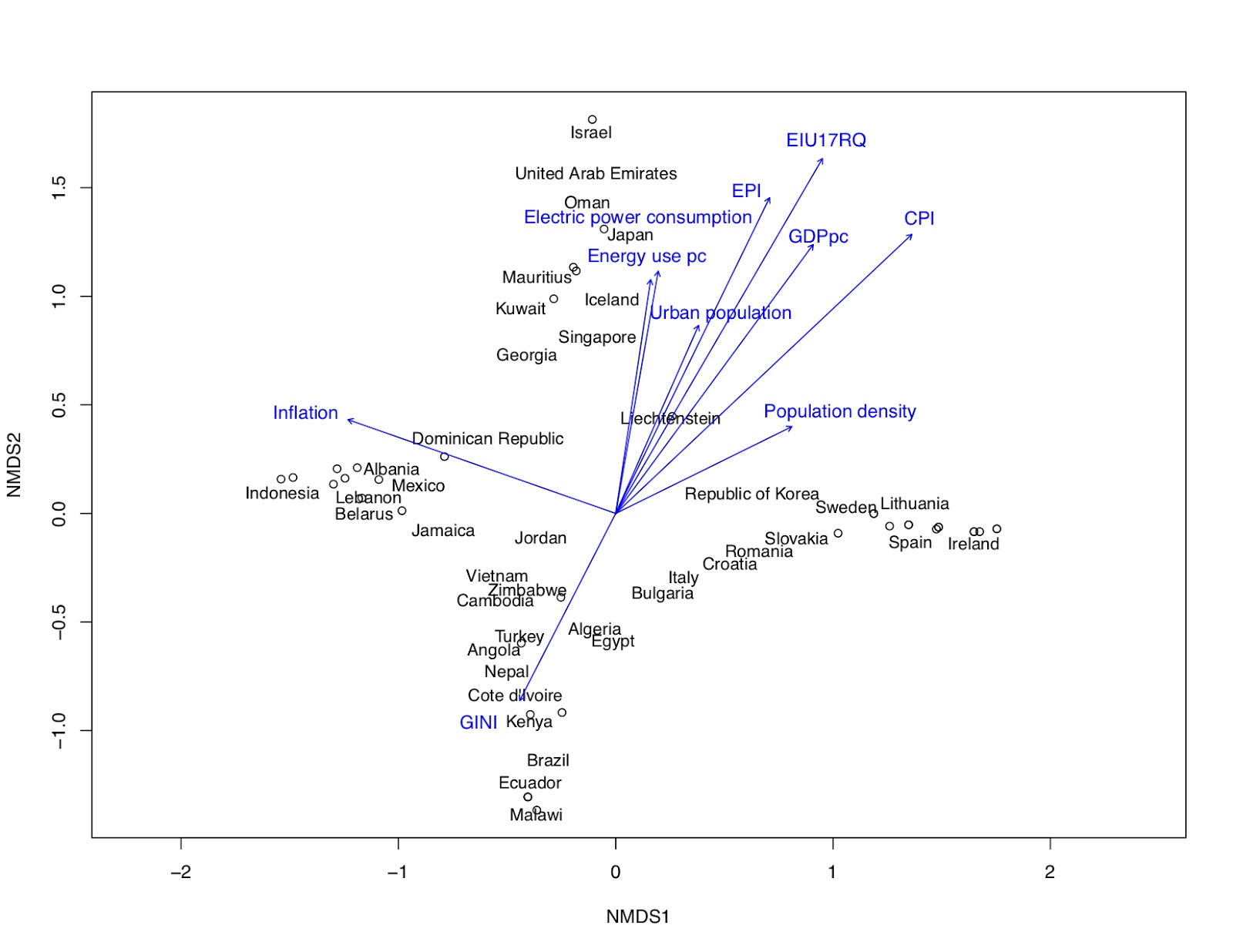


**Figure S4.** Non-metric multidimensional scaling of wild bird demanders, from 2006-2017 (after the EU ban) with additional predictors correlated with the fuzzy clustering of countries in each scenario. 

**Results on projected amount of birds traded**


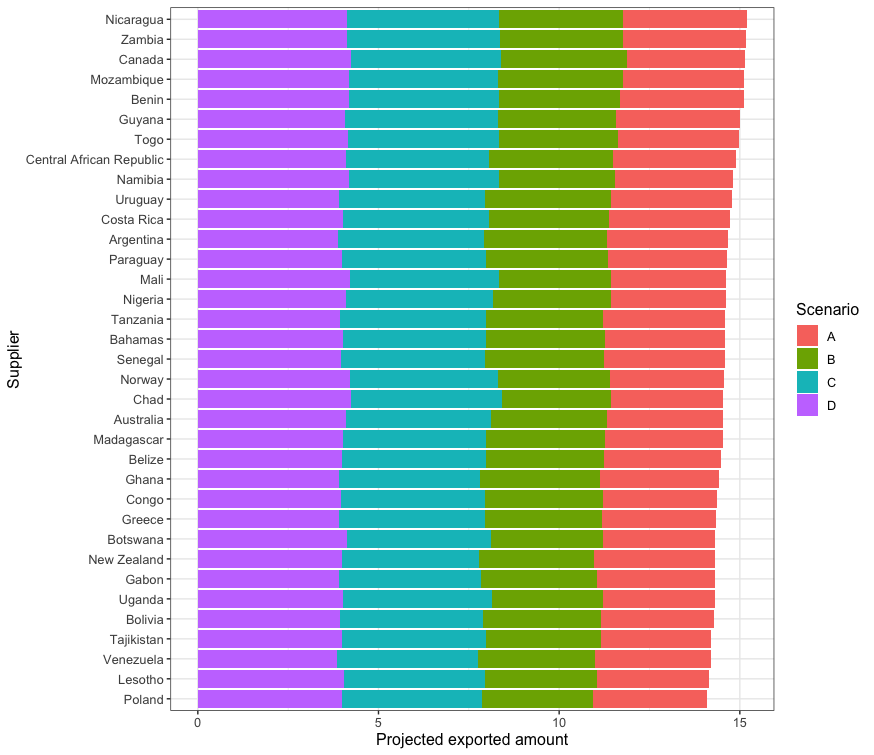

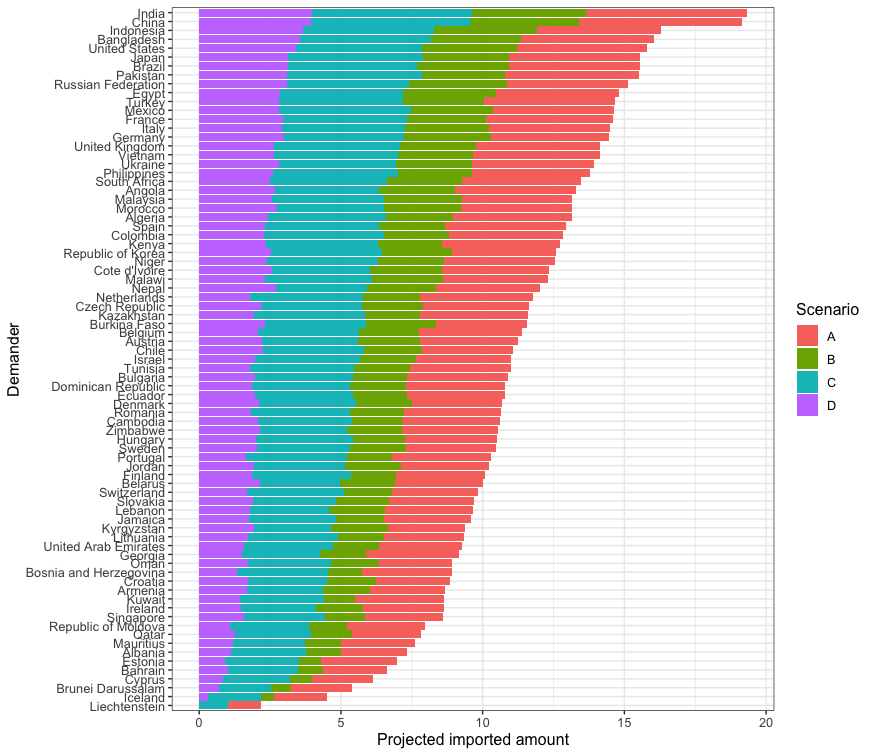


**Figure S5.** Projected amount of birds (log transformed, to improve graphical representation) traded by each demander (upper panel) and supplier country (lower panel) in scenarios A-D considered in this study. Projections were made using the ordinal regression models fit between predicted and actual scores of suppliers and demanders after the EU ban (2006-2017) (**Fig. 5**). Scenario A-Strong trade barriers and rule of law. Scenario B- Strong trade barriers and law enforcement for suppliers, but weak for demanders. Scenario C- Strong trade barriers and law enforcement for demanders, but weak for suppliers. Scenario D-Weak trade barriers and rule of law.


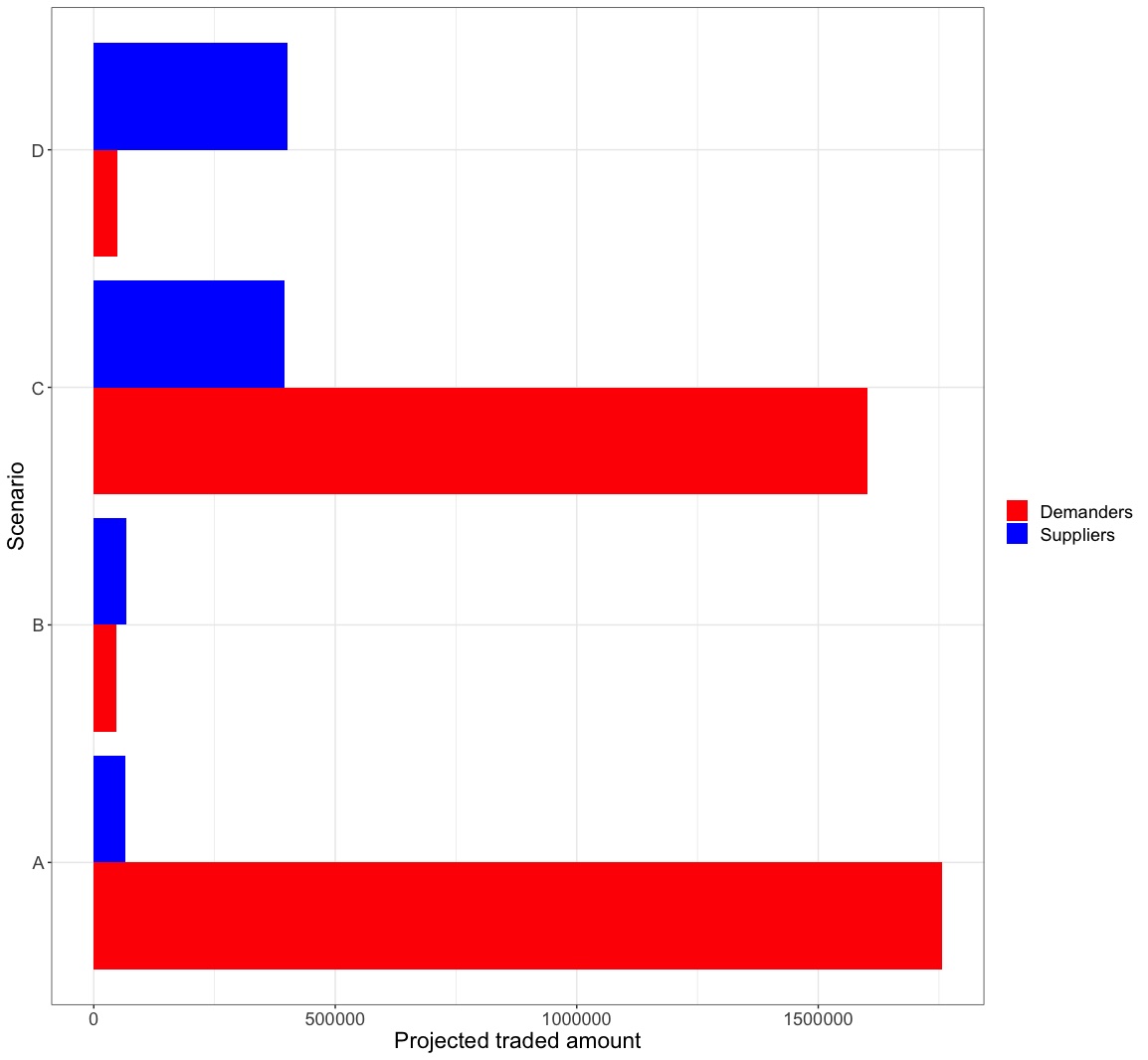


**Figure S6.** Amount of wild birds projected to be traded for each scenario, with amount supplied and demanded. Projections were made using the ordinal regression models fit between predicted and actual scores of suppliers and demanders following the EU ban.

**Supplementary References**

Balsalobre-Lorente D, Shahbaz M, Roubaud D, Farhani S. 2018. How economic growth, renewable electricity and natural resources contribute to CO2 emissions? Energy Policy 113: 356-367.

Bezdek JC.1974. Cluster validity with fuzzy sets. J. Cybernetics, 3: 58-73.

Carrete M, Tella JL. 2008. Wild-bird trade and exotic invasions: A new link of conservation concern? Frontiers in Ecology Environment 6: 207–211. doi: 10.1890/070075

Wyatt T, Johnson K, Hunter L, George R, Gunter R. 2018. Corruption and wildlife trafficking: three case studies involving Asia. Asian Journal of Criminology 13(1): 35-55.

Stern D I. 2012. Modeling international trends in energy efficiency. Energy Economics 34(6): 2200-2208.

Wendling Z A, Emerson J W, Esty D C, Levy M A, de Sherbinin A, et al. 2018. 2018 Environmental Performance Index. New Haven, CT: Yale Center for Environmental Law & Policy. <https://epi.yale.edu/>

Chakrabarti A. 2000. Does trade cause inequality?. Journal of Economic Development 25(2): 1-22.

Masanja G. F. 2014. Human population growth and wildlife extinction in Ugalla ecosystem, western Tanzania. Journal of Sustainable Development Studies 5(2).

Spear D, Foxcroft L C, Bezuidenhout H, McGeoch M A. 2013. Human population density explains alien species richness in protected areas. Biological Conservation 159: 137-147.

Ngwakwe C C, Mokgalong N M. 2014. Consumer income growth and rhino poaching in South Africa. Environmental economics 5(3): 42-52.
