# Supplementary material for: Exploring the Effects of Geopolitical Shifts on Global Wildlife Trade
Source: Bioscience. 2022 Apr 6;72(6):560–72. doi: 10.1093/biosci/biac015 (PMC9180917; doi:10.1093/biosci/biac015)
Supplement: biac015_Supplemental_Files [file biac015_supplemental_files.zip › Original_vs_Logtransformed.docx]

**
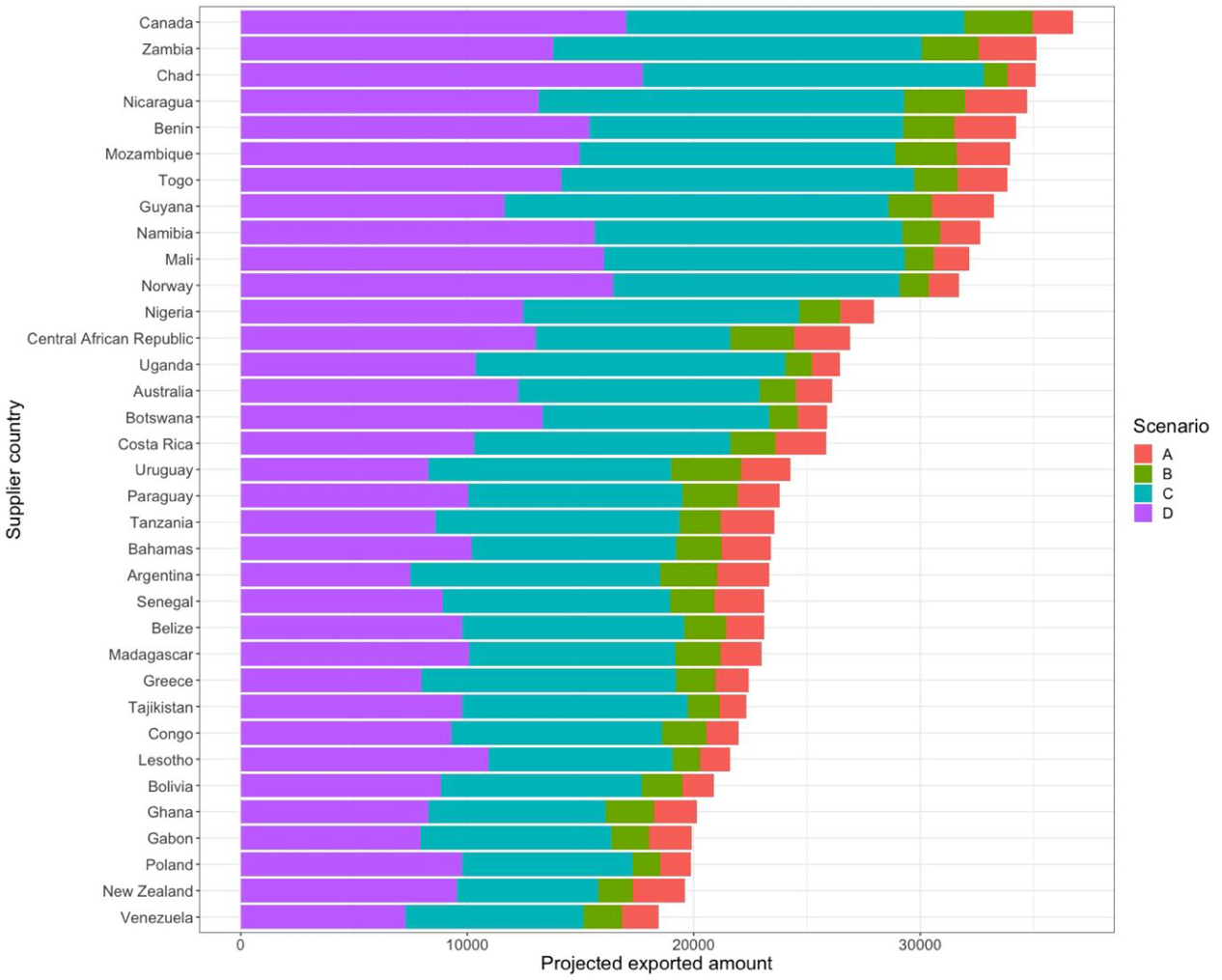

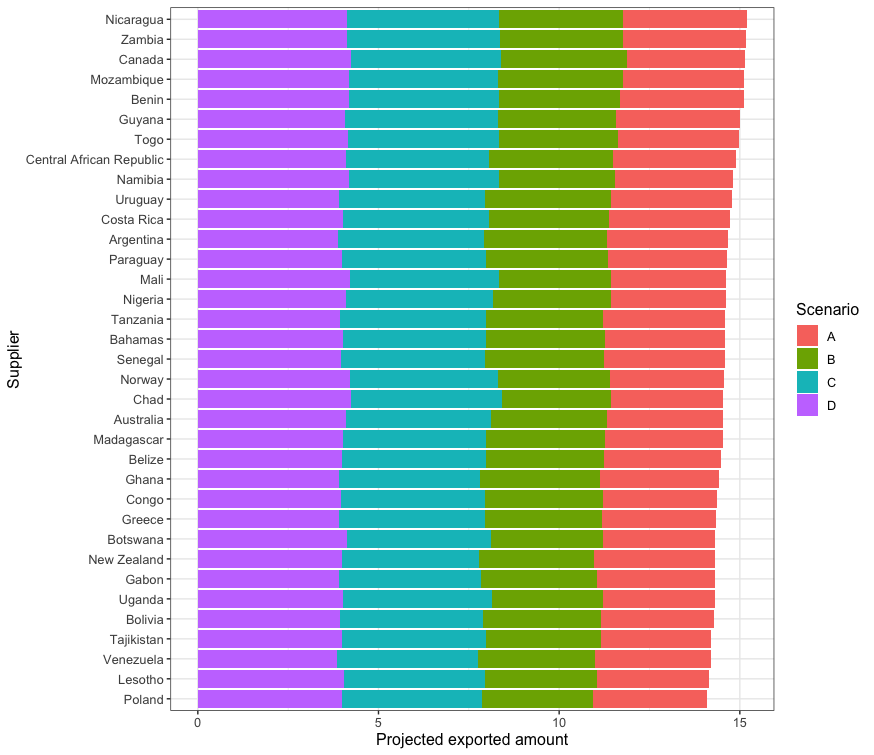
**Projected amount of birds traded by countries classified as suppliers: Original vs log-transformed (regarding **Rev4Com16)**

**Original**

**Log-transformed**
